# Utilization of insecticide treated nets during pregnancy among postpartum women in Ibadan, Nigeria: a cross-sectional study

**DOI:** 10.1186/1471-2393-12-21

**Published:** 2012-03-29

**Authors:** Joel O Aluko, Abimbola O Oluwatosin

**Affiliations:** 1 Nurse/Midwife/Public Health Nurse Tutors Programme, University College Hospital, Orita-mefa, Ibadan, Nigeria; 2 Department of Nursing, Faculty of Clinical Sciences, College of Medicine, University of Ibadan, Nigeria

## Abstract

**Background:**

Pregnant women are susceptible to symptomatic malaria due to invasion of the placenta by plasmodium. Malaria increases the risk of adverse pregnancy outcomes for mothers, the foetuses and newborns. The effective use of Insecticide Treated Nets (ITNs) would be of benefit to these vulnerable women. Previous studies have focused on prenatal-women but this study sought to explore the actual trend of utilization of the proven strategy across all the pregnancy stages among postpartum women in Ibadan.

**Methods:**

This cross-sectional survey utilized a validated structured questionnaire for data collection. A calculated sample of 335 postpartum women was proportionately recruited from three fee-paying facilities within Ibadan, Nigeria using a simple random sampling technique. These hospitals have high client flow for maternity cases and are known for provision of care under traditional ANC model. The data collected were analyzed using descriptive and inferential statistics by means of Statistical Package of Social Sciences (SPSS) version 15. The level of significance was set at = 0.05.

**Results:**

The women's age ranged between 18 and 47 years, mean age was 29.4 ± 0.8 years. Various irregularities marked the traditional model of ANC provided at the settings and no exposure to preconception care. Also, 276 (82.4%) had heard of ITNs. Antenatal clinics formed the major source of information. Low utilization and compliance rates were observed. One hundred and twenty-seven (37.9%) of the women had high knowledge of Malaria in Pregnancy (MIP) but only 70 (20.9%) demonstrated positive attitude towards the use of ITNs. Participants' educational status, family types, employment and residential areas significantly influenced ITNs utilization.

**Conclusions:**

The women knew and learned about ITNs from ANC visits. Majority of the women did not own ITNs because of lack of access to free distribution. The existing traditional model of ANC was marked by irregularities and none of the women was exposed to preconception care. In addition, negative attitude in spite of increased knowledge of MIP was observed among the women. Therefore, evaluation of free distribution of ITNs is recommended. Integration of focused ANC and preconception care are advocated to promote early access to health information.

## Background

Malaria threatens the lives of 3.2 billion people globally and exacts a great toll on vulnerable pregnant women and children, killing an estimated 1 to 2 million yearly and causing illness in about 300 to 500 million among the two groups due to their high vulnerability [1-3].

Africa is a malaria-endemic region where approximately 25 million women become pregnant annually and are at risk of *Plasmodium Falciparum *infection during pregnancy. This factor contributes to the maternal and neonatal morbidity/mortality in African sub-region [4]. Nigeria is one of the hardest hit of the countries in malaria-endemic countries of Sub-Saharan Africa, where the disease accounts for 11% of maternal mortality and 12 to 30% of mortality among the under-five but its severity and complicated effects are most common among infants and pregnant women [1,5]. The severity of malaria is worsened by pregnancy as a result of distinct malarial parasites that bind to the placental [6]. The prevalence of parasitaemia appears greatest in the second trimester and susceptibility to clinical malaria may persist into early postpartum period.

Malaria infection during pregnancy poses substantial risk to the mother, her foetus, and neonate [1]. Apart from the high maternal and infant mortality associated with the disease, it leads to delivery of premature infants and low birth weights due to intrauterine growth retardation (IUGR) resulting from placental parasitisation [1,3]. Hence, its enormous physical, emotional, social and economic impacts on the clients, families and the nation at large cannot be quantified. In 2004, about one-quarter of Nigerian pregnant women were found to have malaria parasites in their blood [5]. Nigerian pregnant women are expected to have acquired immunity due to the high endemic and transmission rates of malaria except the placental form, which they never encounter until they are pregnant. The primigravidae are more susceptible to malaria infection than multigravidae, because the former are still in the process of acquiring natural immunity to placental malaria [6]. Adverse effects that may result from malaria infection on both the mother and child include anaemia in pregnancy, low birth weights, pre-term deliveries, stillbirths and perinatal mortality (in either mother or child or both).

Since the beginning of this century, several attempts have been made to prevent and control the parasitic infection in Nigeria. The Roll Back Malaria (RBM) is the overall national strategy adopted to combat malaria. The RBM strategy seeks to establish a social movement in which the local communities, public and private sectors, all tiers of government and non-governmental agencies come together in a partnership and network to implement malaria control activities. Pregnant women are part of key target groups of RBM. The four key intervention strategies of RBM, which is recommended by the WHO are: case management of malaria in pregnancy, using Sulfadoxine + pyrimethamine (SP) as a drug of choice for intermittent preventive treatment (IPT), widespread use of insecticide treated nets (ITNs) and antenatal care (ANC) [7]. ITNs are promising tools to combat malaria in the country and could be beneficial to women during pregnancy particularly in areas where environmental sanitation has become a serious problem beyond individual manageable capacity.

ITNs have been known to reduce numbers of infective mosquito bites by 70 to 90% in various geographical settings [8]. In Nigeria, it remains a thing of concern that despite the proven efficacy of this preventive intervention and its supposed availability, malaria still constitutes a serious threat to maternal and neonatal health. The 2008 Nigeria Demographic and Health Survey (NDHS) reported that pregnant women who slept under ITNs were only 3% in South-West, Nigeria [9]. This survey provided baseline information for the current study. However, the current study reveals the utilization pattern of ITNs throughout the entire pregnancies (i.e. from conception to delivery). Thus, the study examined the utilization of ITNs during prenancy among postpartum women. The Social Cognitive Theory by Bandura, 1996 [10] was adapted to guide the study.

## Methods

### Design and settings

This cross-sectional descriptive survey assessed the utilization of ITNs during pregnancy among postpartum women attending postnatal and child welfare clinics of the University College Hospital, Adeoyo Memorial Maternity Specialist Hospital and Saint Mary Catholic Hospital (all in Ibadan, Nigeria). Ibadan is the largest city in West African sub-region. The three settings operate the usual traditional model of ANC but have high client flow for maternity.

### Instrument and data collection

The study utilized a validated self-administered questionnaire for data collection. The information elicited includes participants' sociodemographic variables, utilization pattern of ANC, knowledge of MIP and its complications, awareness and use of ITNs and participant's attitude towards ITNs use. The face and content validity of the instrument were ensured by comparing its items with previous studies and by matching them with the stated objectives and formulated research hypotheses. In addition, copies of the research proposal and the instrument went through series of expert reviews in the Department of Nursing, University of Ibadan, Nigeria and UI/UCH Ethical Commitee. In order to ensure reliability of the instrument, the local language (Yoruba) version was developed using 'back-to-back' translation method. Subsequently, a test-retest of the instrument was done prior to the actual data collection exercise. The reliability coefficient was 0.7.

### Population and sampling technique

A sample size of 346 immediate postnatal women (i.e. women whose babies were within the age range of 1 - 42 days or 6 weeks at the instance of data collection) were recruited proportionately from a population of 860 women utilizing the three study settings. The immunization records of each setting served as sample frames. Sample interval (K) was calculated to be 3 using a statistical formula K = N/n, where K = sample interval, N = total population in the sample frame, n = sample size. Thus, every third person on the sample frame was selected subsequent to random selection of number 2 on the list as the starting point on the frame. The study protocol was approved by the UI/UCH Ethical Committee (NHREC/05/01/2008a). Participants were recruited into the study after informed consent has been obtained from each of them.

### Statistical analytical methods

Descriptive and inferential statistics were used for data analysis with the aid of the Statistical Package of Social Sciences (SPSS) version-15. The set hypotheses were tested by using Pearson chi-square analysis association among variables of interest. Level of significance was reported at 5% probability level. The levels of knowledge of MIP among the respondents were measured on a 14-item self-structured scale. For each question item on the scale participants were required to choose one among three options (i.e. "yes", "no", "I don't know"). Questions answered correctly attracts 2 points, "I don't know" attracts 1 point while wrong answers attracts 0 (zero). Maximum obtainable score = 28. The total score of individual respondent was calculated thus:

Respondent's score/Maximum obtainable score x 100 and classified into categories as follows: 80 - 100% = High, 60 - 79% = Average, 0 - 59% = Low. Similarly, the attitudes of the respondents were measured on a 11-item Likert's scale. Respondents were required to choose among 5 options (i.e. "strongly agree", "agree", "undecided", "disagree", "strongly disagree"). These responses attract 1, 2, 3, 4, 5 point(s), respectively. Minimum and maximum scores were 11 and 55, respectively. The total score of individual respondent on this scale was presented in percentage thus: Respondent’s score/Maximum obtainable score × 100 and categorized as follows: 20 - 59 (Negative), 60 - 79 (Uncertain), 80 - 100 (Positive).

## Results

Out of 346 questionnaires administered to participants, 335 were found suitable for analysis. Hence, the response rate was 96.8%. The women's age ranged from 18 to 47 years; mean age being 29.4 ± 0.8 (Table 1). Two hundred and sixty-nine (80.3%) of the women had minimum of senior secondary school education while 220 (65.7%) of the women were self-employed (Table 1). Two-hundred and forty-eight (74.0%) and 67 (20.0%) of the women were residing in high-density areas and slums within Ibadan, respectively (Table 1).

**Table 1:** Participants' socio- demographic characteristics

A	Maternal Age Group (Years)	N	%
	18 - 19	3	0.9
	20 - 29	169	50.5
	30 - 34	101	30.1
	35 - 47	62	18.5
	**Total**	**335**	**100**
**B**	**Levels of Educational**	**N**	**%**
	No formal education	8	2.4
	Primary School Education	39	11.6
	Junior Secondary School (JSS) 1- 3	19	5.7
	Senior Secondary School (SSS) 1- 3	138	41.2
	Tertiary Education	131	39.1
	**Total**	**335**	**100**
**C**	**Employment status**	**N**	**%**
	Applicants	11	3.3
	Students	28	8.4
	Housewives	11	3.3
	Self-employed	220	65.7
	Junior civil servants	13	3.9
	Senior civil servants	38	11.3
	Private staff	14	4.2
	**Total**	**335**	**100**
**D**	**Marriage type**	**N**	**%**
	Monogamy	274	81.8
	Polygamy	47	14.0
	Single (unmarried)	14	4.2
	**Total**	**335**	**100**
**E**	**Ressidential areas**	**N**	**%**
	High density area	248	74.0
	Low density area	20	6.0
	Slum	67	20.0
	**Total**	**335**	**100**

None of the women accessed preconceptional care prior to their immediate past pregnancies. However, 315 (94.0%) of them received antenatal care under the existing traditional model of ANC during their immediate past pregnancies but their visits were marked by irregularities.

### Awareness and utilization of ITNs among the participants

Out of 335 women, 276 (82.4%) had heard of ITNs before the data collection. Antenatal clinics topped the list of major sources of ITN infomation to the women (Table 2). Similarly, 107 (32.0%) had never seen ITNs as at the time of data collection. One hundred and forty-eight (44.2%) women owned ITNs (Table 3). Out of the 148 women who owned ITNs, 12 (8.1%) became ITN owners through free distribution. The major reason for not owning ITNs among non-owners (55.7%) was inaccessibility to free distribution.

**Table 2:** Sources of information of ITNs to participants

Sources of information	N	%
ANC clinics	160	58.0
Electronic media	63	22.8
Printed media	7	2.5
Friends/Neigbours/relations and co-staff	42	15.2
Fellow pregnant women	4	1.5
**Total**	**276**	**100**

**Table 3:** Ownership of ITNs among participants

**A**	**Participants' age group (years)**	**Owners**		**Non-owners**		**Level of significance**
		**N**	**%**	**N**	**%**	
	18 - 19	1	0.7	2	1.1	Chi-sq. = 4.381, df = 3, p-value = 0.223
	20 - 29	67	45.3	102	54.5	
	30 - 34	53	35.8	48	25.7	
	35 - 47	27	18.2	35	18.7	
	**Total**	**148**	**100.0**	**187**	**100.0**	
**B**	**Participants' marriage type**	**N**	**%**	**N**	**%**	**Level of significance**
	Monogamy	134	91.2	150	81.1	Chi-sq. = 8.924, df = 2, p-value = 0.12
	Polygamy	12	8.2	35	18.9	
	No response	1	100.0	0	0.0	
	**Total**	**147**	**100.0**	**185**	**100.0**	
**C**	**Participants' level of education**	**N**	**%**	**N**	**%**	**Level of significance**
	No formal education	2	1.4	4	2.2	Chi-sq. = 24.132, df = 4, p-value = 0.000
	Primary education	11	7.5	28	15.1	
	Junior secondary education	5	3.4	14	7.5	
	Senior secondary education	50	34.0	88	47.3	
	Tertiary education	79	53.7	52	28.0	
	Total	147	100.0	186	100.0	
**D**	**Participants' religion**	**N**	**%**	**N**	**%**	**Level of significance**
	Christianity	86	58.5	84	44.9	Chi-sq. = 6.095, df = 2, p-value = 0.047
	Islam	60	40.8	101	54.0	
	Tradition	1	0.7	2	1.1	
	**Total**	**147**	**100.0**	**187**	**100.0**	
**E**	**Participants' residential areas**	**N**	**%**	**N**	**%**	**Level of significance**
	Densely populated	115	79.3	133	71.9	Chi-sq. = 6.037, df = 2, p-value = 0.049
	Low densely populated	11	7.6	9	4.9	
	Slum	19	13.1	43	23.2	
	**Total**	**145**	**100.0**	**185**	**100.0**	

Table 3 shows that women from monogamous families are more likely to own ITNs than their counterparts who are from polygamous families. This was found to be significant (Chi-square = 8.92, df = 2, p. value = 0.012). In addition, women with high level of formal education background are more likely to own ITNs than their counterparts with low or no formal educational background (Chi-square = 24.13, df = 4, p-value = 0.000). Furthermore, women who are from Christian backgrounds are more likely to own ITNs than their counterparts who are from other religions. This was also found to be significant; (Chi-square = 6.095, df = 2, P value = 0.047). Besides, women who live in areas of low population density are more likely to own ITNs than those who live in areas of high population density, while those who live in areas of high population density are more likely to own ITNs than those who live in slums. This was found to be significant (Chi-square = 6.037, df = 2, P value = 0.049).

**Figure 1:**
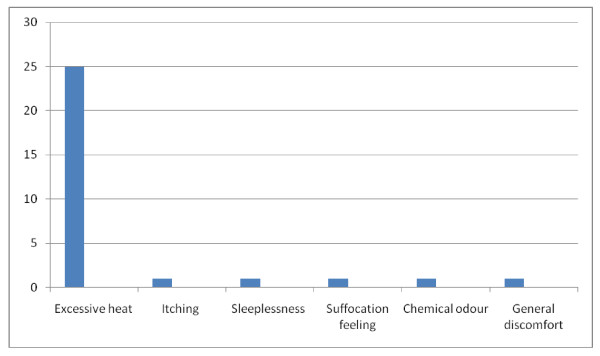
**Nature of discomfort experienced by ITN users**.

**Figure 2:**
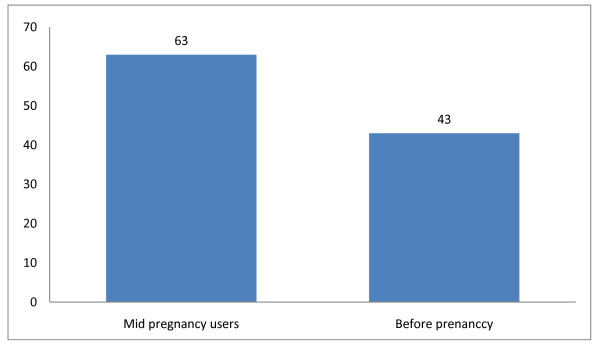
**Timing of commencement of ITNs use among users**.

Out of the 148 women who owned ITNs, 106 (71.6%) of them slept under the nets during their immediate past pregnancies; that is, 31.6% utilization rate among the 335 women studied. Sixty-three (59.4%) among the 106 ITNs users claimed to have slept under them daily; this is equivalent to 18.8% compliance rate. Out of the 106 women who slept under ITNs during their immediate past pregnancy, 30 (28.3%) of them experienced at least a kind of discomfort. Among the women who experienced discomfort, those who experienced excessive heat while sleeping under ITNs were 25 (83.3%) (Figure 1). It is important to note that out of the 106 women who slept under ITNs during their immediate past pregnancies, 63 (59.4%) of them commenced the use mid pregnancy (Figure 2).

Similar to ownership of ITNs above, the study showed that women from monogamous families are three-times more likely to sleep under ITNs than their counterparts who are from polygamous families. This was found to be significant (Chi-square = 9.65, df = 2, p. value = 0.008). Besides, women with good formal education background are more likely to sleep under ITNs than their counterparts with no or low level of formal educational background (Chi-square = 13.52, df = 4, P value = 0.009). In contrast, women who are dependants are more likely to sleep under ITNs than their counterparts who are employed (Chi-square = 23.23, df = 9, P value = 0.006).

### Knowledge of MIP among participants

**Figure 3:**
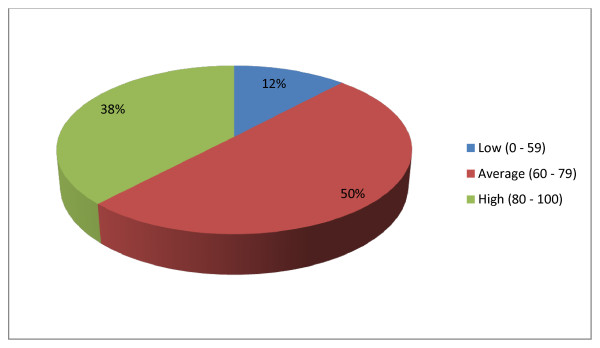
**Participants' level of knowlege of MIP (with the ratings)**.

One-hundred and sixty-eight (50.1%) and 127 (37.9%) of the participants had average and high knowledge of MIP, respectively (Figure 3).

### Attitudes of participants towards use of ITNs

**Figure 4:**
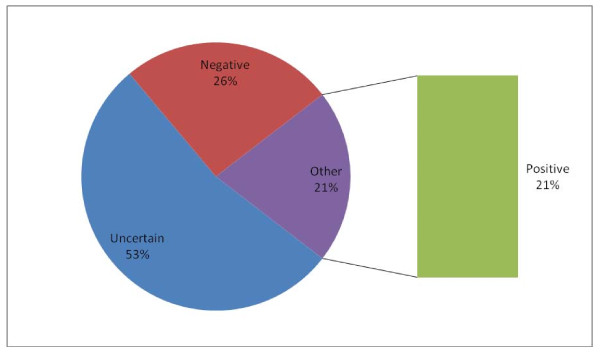
**Attitudes of participants towards ITNs use**.

The women showed different kinds of attitudes towards utilization of ITNs in their responses: while 179 (53.4%) were uncertain, 86 (25.7%) showed negative attitude which did not favour the utilization of ITNs (Figure 4).

## Discussion and conclusions

The participants' mean age was 29.4 years ± 0.8. This is expected because this is representative of the pregnant women in Nigeria. Other studies within this geo-political zone and elsewhere confirm this finding [11-13].

The women studied knew about ITNs. Majority learned about the ITNs during ANC. This finding confirmed the efficacy of regular health education/information the women accessed during antenatal clinic visits. The practice of disseminating health information is the norm in all formal health facilities in the country and such should be encouraged. This finding agrees with that of Abasiattai (2009) and Karunamoorthi et al. (2010) in which acquisition of health information was attributed to ANC [14,15]. However, majority of the women did not own ITNs. This is because they were unable to access the material through the free ITNs distribution programme. This is not a good development and it therefore requires attention. In order to solve the problems it has been suggested that the material should be provided through antenatal clinics (both private and public) [16,17]. Surprisingly, ITN utilization and compliance rates were only 32% and about 19%, respectively. Although, previous studies [11,12] reported lower utilization and compliance rate, the current situation still requires urgent intervention from stakeholders particularly, the Federal Government. In addition, those who commenced sleeping under ITNs before getting pregnant were fewer than 15%. This is because there are no organized preconception care and most pregnancies even among the married may not be planned. This perhaps was responsible for not commencing sleeping under ITNs prior to conception. By the time women come for ANC they have contracted pregnancy malaria, which have already infiltrated the placenta. In addition, the negative attitudes of the women towards ITN use must have contributed to low utilization and compliance rates. However, these factors among others were likely responsible for increased episodes of malaria attacks recorded in this study. Therefore, it is imperative to encourage regular, early and daily use of ITNs before and during pregnancy among the women. It is believed that sleeping under ITNs before conception and throughout the entire period of pregnancy will certainly reduce incidence of MIP in the society.

It is necessary to point out that more than a quarter of the women who slept under ITNs experienced at least one form of discomfort, excessive heat being the major discomfort. This is probably because of the nature of the hot weather, typical of Africa. Regular electricity supply to power electric fans and airconditioners will likely improve ITN utilization and compliance rate. The government should look into this aspect because regular power supply contributes greatly to effective delivery and utilization of health care products.

Very few women showed positive attitudes towards utilization of ITNs. It is a thing of concern that their relative high knowledge of MIP did not inform high level of positive attitude towards the use of ITNs in particular. This unfavourable finding requires further studies. Akinleye et al. (2009) reported similar finding among women in rural South-West of Nigeria [18]. In addition, the reported side effects of ITNs on its users might have contributed to the demonstration of the negative attitudes earlier mentioned. This explanation is supported by a study carried out by Felema (2007) [19].

In order to describe in a better way the women who are likely to own ITNs we analyzed the data for marriage type, education, religion and occupation. This is because certain sociodemographic factors often influence the utilization of health care services or products. The findings of the analysis are not without certain implications. First, women who were from monogamous families were more likely to own and sleep under ITNs than their counterparts in polygamous family. This implies that a woman who is the only wife of her husband is more likely to get necessary social and financial supports from the husband. Secondly, women who had at least senior secondary school education are more likely to own ITNs than their counterparts with lower level of education. This reinforces the importance of girl-child education as a means of women empowerment.

Thirdly, women who are dependants are more likely to sleep under ITNs than their counterparts who engage in at least one type of employment. This is surprising! This could be due to the low earnings of the people as reported among the women in this study. This finding differs from what Yusuf et al. (2008) found in their study, in which there was a rarity of malaria preventive measures among the dependants (i.e. students, housewives and unemployed) [12]. Fourthly, women from Christian backgrounds are more likely to own and sleep under ITNs than their counterparts from other religious backgrounds, though the later was not significant. Although, polygamy is more favoured in Islam than Christianity, yet further studies may explore this area in future.

Moreover, women who live in low-density areas are more likely to own and sleep under ITNs than their counterparts living in high-density areas. Similarly, those living in high-density areas are more likely to own and sleep under ITNs than those living in slums. The fact remains that, while people of low socio-economic status live in high-density areas or slums, the 'well-to-dos' usually live in low-density areas where modern apartments exist.

In conclusion, majority of the women did not own ITNs and had not benefit from the free distribution of ITNs programmes due to inaccessibility. Besides, some women did not like sleeping under these ITNs because they experienced some discomforts, mostly excessive heat that could be prevented by regular power supply. In addition, late initiation and irregular clinic visits characterized the practiced traditional model of ANC and the women had no access to preconception care before pregnancy which might likely be responsible for low ITN compliance rate resulting from delayed access to information on prevention of MIP with ITNs. Moreover, negative attitudes towards utilization of ITNs in spite of increased level of knowlege of MIP were implicated for non-use. Therefore, it is recommended that the free ITNs distribution programme of the Federal Government of Nigeria should be examined and reviewed for effectiveness. In addition, the proven focused antenatal care and preconception care should be integrated into the existing health care system in the country. This is because both care would likely aid early access to relevant health information/education and form easy avenues where free distribution of malaria prevention materials could be effectively carried out.

### Limitations of the study

Although, this study has been able to examine ITNs use throughout the entire period of pregnancy, the data collected was based on ability of the women to recall events of the last nine months of pregnancy.

## Abbreviations

WHO: World Health Organization; ITNs: Insecticide treated bednets; MIP: Malaria in pregnancy; ANC: Antenatal care; NDHS: Nigeria demographic health survey; MPM: Malaria preventive model; SCT: Social cognitive theory; IUGR: Intrauterine growth retardation; RBM: Roll back malaria; FMOH: Federal ministry of health; SPSS: Statistical package of social sciences; SP: Sulfadoxine + pyrimethamine.

## Competing interests

The authors declare that they have no competing interests.

## Authors' contributions

AJO conceived and designed the study, developed the data collection instruments, supervised data collection, performed the statistical analysis, and wrote the draft of the manuscript. OOA participated in the study design, advised on data analysis, reviewed and finalized the data collection instrument, corrected the draft of the manuscript and contributed to the manuscript. All authors read and approved the final manuscript.

## Pre-publication history

The pre-publication history for this paper can be accessed here:

http://www.biomedcentral.com/1471-2393/12/21/prepub
